# Al_2_O_3_ Disk Supported Si_3_N_4_ Hydrogen Purification Membrane for Low Temperature Polymer Electrolyte Membrane Fuel Cells

**DOI:** 10.3390/membranes3040406

**Published:** 2013-12-05

**Authors:** Xiaoteng Liu, Paul A. Christensen, Stephen M. Kelly, Vincent Rocher, Keith Scott

**Affiliations:** 1 School of Chemical Engineering and Advanced Materials, Merz Court, Newcastle University, Newcastle upon Tyne NE1 7RU, UK; E-Mails: paul.christensen@ncl.ac.uk (P.A.C.); k.scott@ncl.ac.uk (K.S.); 2 Department of Chemistry, University of Hull, Cottingham Road, Hull HU6 7RX, UK; E-Mails: s.m.kelly@hull.ac.uk (S.M.K.); vincent.rocher@espci.org (V.R.)

**Keywords:** Al_2_O_3_ disk supported Si_3_N_4_ membrane, hydrogen purification, CO removal, fuel cells, Pt catalyst

## Abstract

Reformate gas, a commonly employed fuel for polymer electrolyte membrane fuel cells (PEMFCs), contains carbon monoxide, which poisons Pt-containing anodes in such devices. A novel, low-cost mesoporous Si_3_N_4_ selective gas separation material was tested as a hydrogen clean-up membrane to remove CO from simulated feed gas to single-cell PEMFC, employing Nafion as the polymer electrolyte membrane. Polarization and power density measurements and gas chromatography showed a clear effect of separating the CO from the gas mixture; the performance and durability of the fuel cell was thereby significantly improved.

## 1. Introduction

The hydrogen-fuelled, proton-exchange membrane fuel cell is a promising power source because of its high power density, high-energy conversion efficiency and low emission level. However, it requires high-purity H_2_ when operated at low temperatures, since impurities in the H_2_ stream, such as CO, will poison and therefore reduce the performance of Pt-based anodes. It has been reported that a CO concentration of 10 ppm and above will poison Pt [1,2]. A significant amount of research has been carried out to improve the CO tolerance by using advanced Pt alloy catalysts [3,4,5,6], or alternative membranes capable of operating at higher temperatures [7,8,9,10,11]. H_2_ purifying devices can also be used to remove CO, but the use of such additional steps has a negative effect on the overall process in terms of cost and efficiency [12,13].

The low-cost mesoporous silicon nitride (Si_3_N_4_) employed in this study, has a high surface area (≥400 m^2^ g^−1^, determined using the Brunauer–Emmett–Teller (BET) method [14]) and a narrow pore-size distribution (20–60 Å) [14,15]. The small pore size and large polar surface area of silicon nitride renders it of possible interest with respect to application as a selective gas filter [16]. The polar nature of the surface sites on silicon nitride also gives rise to complicated physisorption and chemisorption reactions which may influence its gas separation properties [17,18]. A thin mesoporous Si_3_N_4_ layer deposited on top of a microporous Al_2_O_3_ disk support has shown high selective absorption of NO_2_ [15], and also potential application in the separation of CO from reformate.

In the work reported in this paper, the Nafion membrane system was employed as the polymer electrolyte membrane (PEM) in the polymer electrolyte membrane fuel cells (PEMFCs) fuelled by a mix of CO and H_2_ passing through a Si_3_N_4_ membrane. Because it is clear to see the CO poisoning effect significantly reduces such fuel cell’s performance which operates at low temperatures.

## 2. Results and Discussion

### 2.1. Scanning Electron Microscopy

**Figure 1:**
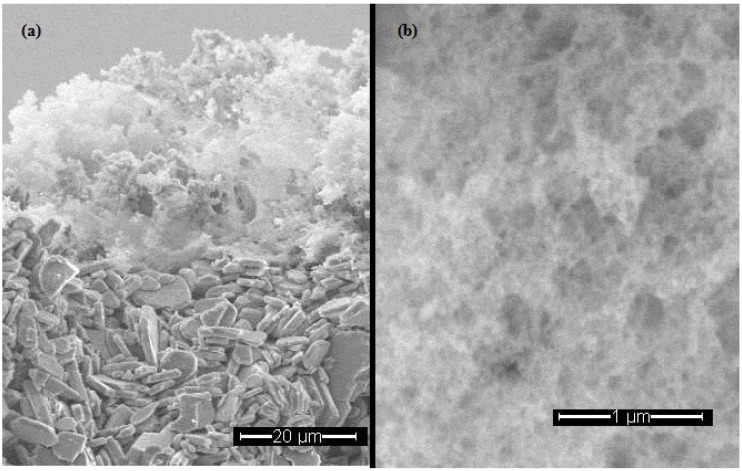
Scanning electron micrographs (SEMs) of the Si_3_N_4_ membrane supported on Al_2_O_3_.

Figure 1a shows a scanning electron micrograph (SEM) image (×1000) of a cross-section of the Si_3_N_4_ membrane supported on Al_2_O_3_. A reasonably uniform Si_3_N_4_ appears on the top of the Al_2_O_3_ disk. As can be seen in the figure, the thickness of the Si_3_N_4_ layer was approximately 20 µm. Figure 1b shows the Si_3_N_4_ layer at higher magnification (×25,000) and it appears from the figure that the Si_3_N_4_ was highly porous and could possibly act as an effective absorbent for CO.

### 2.2. Gas Chromatography (GC)

**Figure 2:**
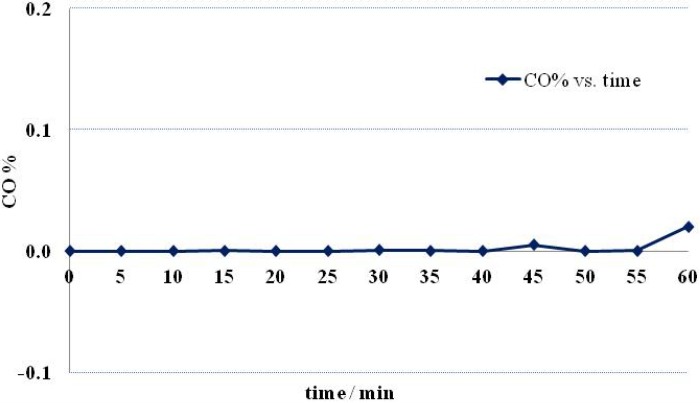
The volumetric concentration of CO in the Si_3_N_4_ membrane-filtered gas as a function of time.

The concentration of CO in H_2_ in the exhaust from the filter H_2_ (CO%) was calculated from the GC spectra. Figure 2 shows CO% as a function of operation time. As may be seen from the figure, CO was not detected in the outlet gas for the first 40 min; at longer times, the CO concentration slightly increased, suggesting the filter may allow a tiny amount of CO to pass through. The separation phenomenon might be via mixed chemisorption and physisorption functions, but it cannot be identified at this stage. The majority of CO was blocked by the membrane and discharged from the bypass outlet. The tiny amount of CO that passed through the membrane may have resulted from the layer of Si_3_N_4_ being too thin. Increasing the amount of such material or packing it in a cartridge may be a possible solution.

### 2.3. Fuel Cell Testing

**Figure 3:**
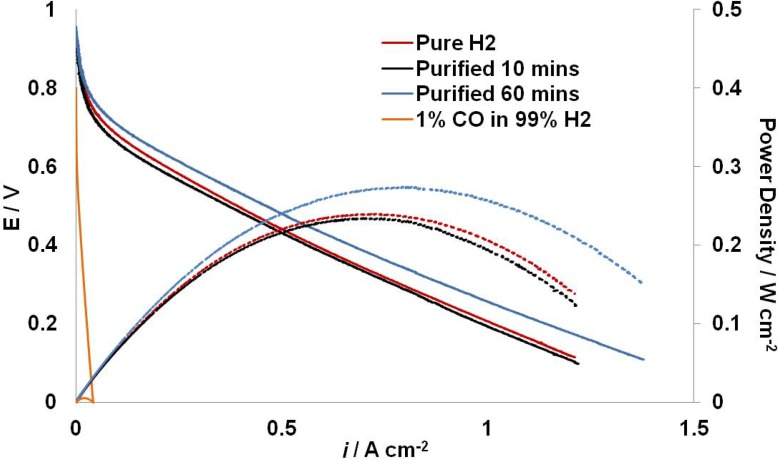
Polarization (solid) and power density (dash) curves of the fuel cell. The blue curve fuel cell with pure H_2_ as the anode feed. The red curves represent the fuel cell with anode feeding gas of filtered mixed gas at 10 min and the black curves represent the curves obtained at 60 min. The orange curves represent the fuel cell performance with anode feeding gas of 1% CO in 99% H_2_.

Figure 3 shows the polarization curves and power density curves of the fuel cell. The fuel cell was operated for 60 min with the filtered gas as anode feeding gas, the forward sweep at 10 min and 60 min are shown. The curve obtained using pure H_2_ as anode feeding gas in the same fuel cell is also included for comparison, as well as the curve obtained using 1% CO in 99% H_2_ as anode feeding gas. The operating temperature of the PBI system was 80 °C.

With pure H_2_, a maximum power density of 0.29 W cm^−2^ was achieved. The 1% CO in H_2_ mixture filtered through the Si_3_N_4_ membrane was then fed into the anode, replacing pure H_2_. The performance of the fuel cell was decreased with a maximum power density 0.24 W cm^−2^. As the bypass value was slightly open, the flow rate after the membrane would be lower than 300 cm^3^ min^−1^; presumably there was a small pressure drop too. This might be the reason for the lower fuel cell performance. However, after another 50 min operation, the fuel cell remained a similar performance with less than 0.01 W cm^−2^ decreases in maximum power density. This is the evidence that the CO concentration of the filtered gas was lower than 10 ppm; the majority of CO was separated by the membrane. However, longer operation time will be needed to verify the separating function of the Si_3_N_4_ membranes.

When the anode feeding gas switched to 1% CO in 99% H_2_, the fuel cell performance became affected from the very beginning, and it showed a complete CO poisoning effect to the anode catalyst.

## 3. Experimental Section

### 3.1. Si_3_N_4_ Membrane

All chemicals were obtained from Sigma-Aldrich (Dorset, UK). The Al_2_O_3_ disks, as the membrane support, were prepared from Al_2_O_3_ powder using water as binder. The disks were then sintered at 1000 °C for 4 h. All procedures were performed under a protective nitrogen atmosphere using standard Schlenk techniques or in a nitrogen-filled glove box. The Si_3_N_4_ membranes were prepared by dipping Al_2_O_3_ support disks into a silicon di-imide sol followed by sintering [15,19]. Briefly, the silicon diimide sol was prepared by adding trifluoromethanesulfonic acid into a solution of TDSA in dry THF. The mixture was heated at 50 °C for 16 h then cool to room temperature. A solution of ammonia in cold THF was then added and the mixture left quiescent at room temperature for 18 h, then ammonia gas was bubbled through the solution for 10 min. After being left quiescent for 1 h, Al_2_O_3_ support disks were dipped in the sol for 10 min and then dried under N_2_ flow. After 30 min the disks were dipped again for another 10 min. The disks were dried under N_2_ flow overnight and then pyrolyzed under NH_3_ flow at 1000 °C for 2 h. The heating rate for the pyrolysis was 2 °C min^−1^. The disks so obtained were 1.6 cm in diameter, 0.1 cm in thickness, so that 4.5 cm^2^ in total surface area. On average, 9 mg of dry Si_3_N_4_ was deposited on each Al_2_O_3_ disk.

### 3.2. Scanning Electron Microscopy (SEM)

Scanning electron micrographs were obtained using a Philips XL30 ESEM-FEG SEM instrument. The Si_3_N_4_ membrane was imaged without any additional coating. The membrane was dipped in liquid nitrogen and then broken using pliers to obtain a clean cross-section.

### 3.3. The Membrane Holder

**Figure 4:**
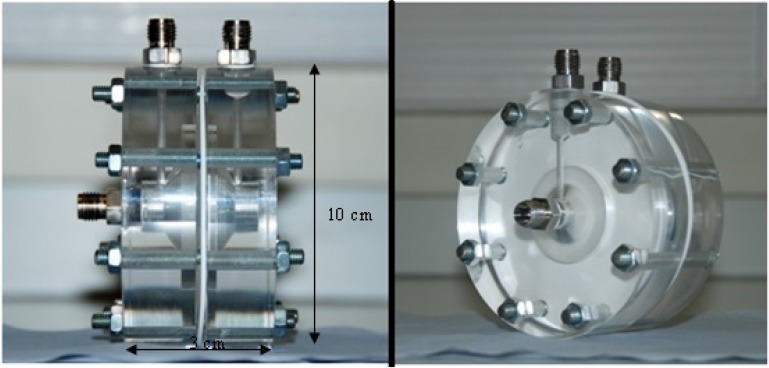
The holder cell for the Si_3_N_4_ membrane.

Figure 4 shows the holder cell for the Si_3_N_4_ membranes. The cell allowed a mixture of various compositions of CO and H_2_ to flow through the membrane at rates up to 300 cm^3^ min^−1^. The two chambers were made from poly(methyl methacrylate), and silicone rubber sheets were employed as gaskets to ensure a good seal between the holder and the Si_3_N_4_ membrane. The edge of the membrane was covered by silicone rubber, and the effective area of the membrane was 1.3 cm^2^. As it has been shown in Section 3.1 that each membrane has a surface area of 4.5 cm^2^, assuming Si_3_N_4_ was homogeneously deposited on the Al_2_O_3_ disk, then 37.6% of the Si_3_N_4_ was responsible for separating the CO. 

### 3.4. The Fuel Cell Test Rig

**Figure 5:**
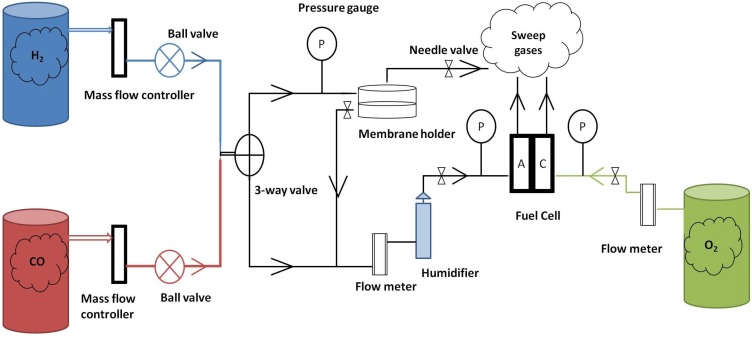
Schematic diagram of the fuel cell rig.

Figure 5 shows a schematic diagram of the fuel cell rig. The mass flow controllers were used to produce 1% CO in 99% H_2_ by volume for fuel cell test and gas chromatography (GC) test. The CO + H_2_ was fed to the Si_3_N_4_ membrane at a flow rate of 300 cm^3^ min^−1^ and then humidified by passing through a humidifier with di-ionized water maintained at 110 °C prior to entering the anode compartment of the PEMFC. The bypass needle value (marked as A in Figure 5) was slightly open to allow filtered CO exhausting and keep neutral pressure before the membrane in the cell. The cathode feed gas was pure oxygen at a flow rate of 300 mL min^−1^. The gas samples for GC were collected before entering the humidifier. All gasses were high purity (99.9%) and purchased from BOC Industrial Gases.

### 3.5. GC

One percent CO in 99% H_2_ mixed gas at the rate of 300 mL min^−1^ was fed to the Si_3_N_4_ and then the exhaust gas samples were monitored using a SHIMADZU GC-8A Gas Chromatograph (Kyoto, Japan) fitted with a Molecular Sieve 5A column.

### 3.6. Fuel Cell and Membrane Electrolyte Assembly (MEA)

**Figure 6:**
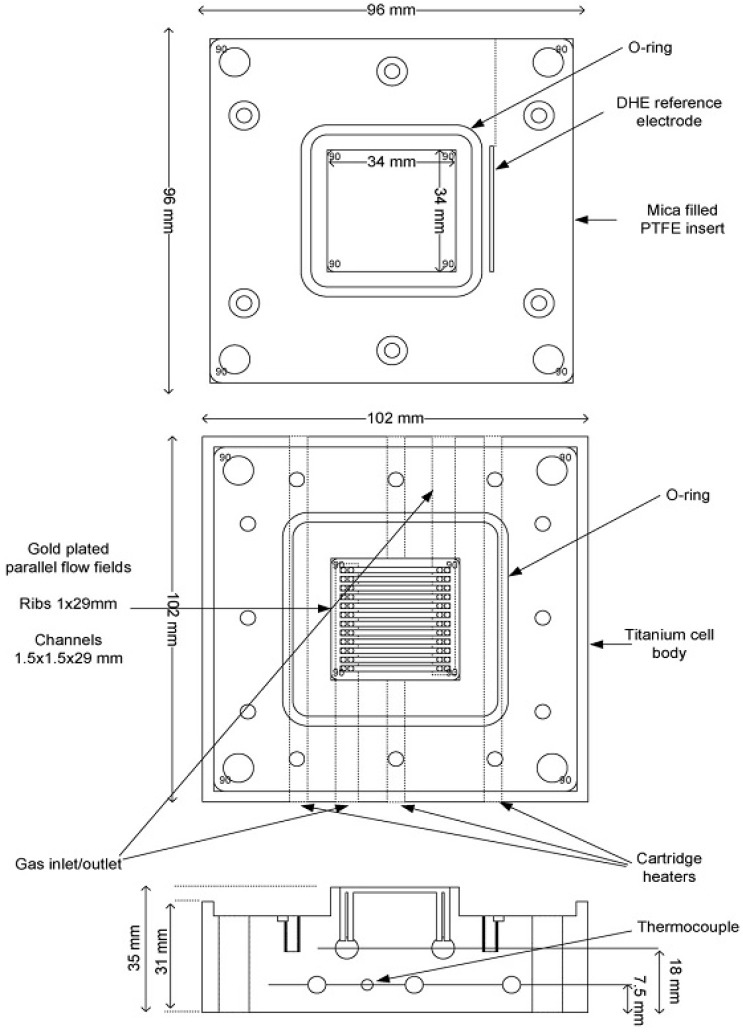
Schematic diagram of the fuel cell.

Details of the fuel cell employed may be found in previous papers [8,9,20]. Figure 6 shows a schematic diagram of the fuel cell. The cell body was made from titanium, the anode and cathode current collectors comprised 3.4 cm × 3.4 cm gold-plated Ti blocks, into which were cut parallel-flow fields to allow gases transport. Mica filled PTFE inserts were used to surround the flow fields and provide location for the O-ring seal and dynamic hydrogen electrodes (DHEs). The DHEs consisted of two platinum wires located on each side of the membrane outside the O-ring. Thermostatically controlled cartridge heaters (6 of 10 mm in diameter 100 mm in length, 150 W, RS, Northants, UK) were inserted into the Ti blocks to control the temperature. A 20 A potentiostat (Sycopel, UK) combined with a high impedance multi-channel data acquisition card (National Instruments, NI6010) was employed to carry out the electrochemical measurements. Polarisation curves were recorded during at a scan rate of 10 mV s^−1^.

The catalysts were purchased from Alfa Aesar. The anode was made from 20% Pt/C (VulcanXC-72) with a Pt loading of 0.2 mg cm^−2^. The cathode was made from 50% Pt/C (VulcanXC-72) with a Pt loading of 0.5 mg cm^−2^. 60% PTFE solution in di-ionized water (Sigma-Aldrich, Dorset, UK) was used as binder. The gas diffusion layer was made of carbon paper (10% GDL, 40% MPL, H2315T10AC) sourced from Freudenberg FCCT KG. Taking the anode preparation as an example, the ink was made by adding a small amount of de-ionized water into PTFE solution (20.4 mg) and mixing in a glass sample holder, followed by ultra-sonication for 15 min. The required amount of Pt/C catalyst (49.0 mg) and isopropanol (130.6 mg) were added to the aqueous PTFE solution, the suspension was then placed in the ultrasonic bath for a further 30 min. The preparation was carried out at room temperature. The carbon paper was heated on a hot plate to maintain the temperature at 80 °C to 100 °C for good liquid evaporation. The ink was sprayed evenly onto the surface of the carbon paper using a Badger Model 100™ spray gun fed by N_2_ gas. The Nafion 117 membrane was sandwiched between the cathode and the anode, and then hot pressed for 3 min at a pressure of 500 kg and temperature of 100 °C.

## 4. Conclusions

It has been shown that an Al_2_O_3_-supported Si_3_N_4_ membrane was capable of separating CO from a mixed stream of CO and H_2_. The separation phenomenon could be achieved via mixed chemisorption and physisorption functions. The separated CO was discharged from the bypass gas outlet so that the pressure in the cell remained the same as the surrounding atmosphere. A pore size of Si_3_N_4_ could also block CO molecules and still allow H_2_ to pass through the membrane. Since Si_3_N_4_ has the advantages of being low cost and with ease of fabrication, it is probable that increasing the amount of Si_3_N_4_ used or changing the fabrication method would further improve the efficiency of the Si_3_N_4_ membrane to clean CO from a stream of H_2_.
